# Genetic Parameters for Yolk Cholesterol and Transcriptional Evidence Indicate a Role of Lipoprotein Lipase in the Cholesterol Metabolism of the Chinese Wenchang Chicken

**DOI:** 10.3389/fgene.2019.00902

**Published:** 2019-10-03

**Authors:** Xingyong Chen, Wenjun Zhu, Yeye Du, Xue Liu, Zhaoyu Geng

**Affiliations:** ^1^College of Animal Science and Technology, Anhui Agricultural University, Hefei, China; ^2^Anhui Province Key Laboratory of Local Livestock and Poultry Genetic Resource Conservation and Bio-breeding, Anhui Agricultural University, Hefei, China

**Keywords:** heritability, lipoprotein lipase, Wenchang chicken, yolk cholesterol, egg quality

## Abstract

The yolk cholesterol has been reported to affect egg quality and breeding performance in chickens. However, the genetic parameters and molecular mechanisms regulating yolk cholesterol remain largely unknown. Here, we used the Wenchang chicken, a Chinese indigenous breed with a complete pedigree, as an experimental model, and we examined 24 sire families (24 males and 240 females) and their 362 daughters. First, egg quality and yolk cholesterol content were determined in 40-week-old chickens of two consecutive generations, and the heritability of these parameters was analyzed using the half-sib correlation method. Among first-generation individuals, the egg weight, egg shape index, shell strength, shell thickness, yolk weight, egg white height, Haugh unit, and cholesterol content were 45.36 ± 4.44 g, 0.81 ± 0.12, 3.07 ± 0.92 kg/cm^2^, 0.340 ± 0.032 mm, 15.57 ± 1.64 g, 3.36 ± 1.15 mm, 58.70 ± 12.33, and 274.3 ± 36.73 mg/egg, respectively. When these indexes were compared to those of the following generation, no statistically significant difference was detected. Although yolk cholesterol content was not associated with egg quality in females, an increase in yolk cholesterol content was correlated with increased yolk weight and albumin height in sire families (*p* < 0.05). Moreover, the heritability estimates for the yolk cholesterol content were 0.328 and 0.530 in female and sire families, respectively. Therefore, the yolk cholesterol content was more strongly associated with the sire family. Next, chickens with low and high yolk cholesterol contents were selected for follicular membrane collection. Total RNA was extracted from these samples and used as a template for transcriptional sequencing. In total, 375 down- and 578 upregulated genes were identified by comparing the RNA sequencing data of chickens with high and low yolk cholesterol contents. Furthermore, Gene Ontology term and Kyoto Encyclopedia of Genes and Genomes pathway enrichment analyses indicated the involvement of energy metabolism and immune-related pathways in yolk cholesterol deposition. Several genes participating in the regulation of the yolk cholesterol content were located on the sex chromosome Z, among which lipoprotein lipase (*LPL*) was associated with the peroxisome proliferator-activated receptor signaling pathway and the Gene Ontology term cellular component. Collectively, our data suggested that the ovarian steroidogenesis pathway and the downregulation of *LPL* played critical roles in the regulation of yolk cholesterol content.

## Introduction

On the day of hatch, most of the yolk sac has been absorbed by the bird, which provides sufficient nutrition for the first days (0–3 days) posthatch (Yair and Uni, 2011). Moreover, it is widely accepted that both growth and breeding performance of birds depend largely on their early health (Yadgary et al., 2010). Therefore, egg yolk quality plays an essential role in maintaining early health and later breeding performance. The main components of the egg yolk are triglyceride, cholesterol, lecithin, vitamins, and minerals (Ding et al., 2017). Previous studies have suggested that cholesterol intake from eggs can affect human health, causing dyslipidemia, hyperlipidemia, atherosclerosis, or cardiovascular diseases (Andersen et al., 2013; Omole and Ighodaro, 2013). Nevertheless, yolk cholesterol is essential for egg production and embryo development. Indeed, in hens that had decreased or insufficient cholesterol synthesis to maintain embryonic development, egg production was reduced or stopped (Janjira, 2017). Furthermore, cholesterol homeostasis is essential and correlates with egg hatchability. While hatchability was increased when the yolk cholesterol content reached a certain level, it was decreased when cholesterol levels increased further and exceeded a certain threshold (Dikmen and Sahan, 2007).

Yolk cholesterol is mainly derived from *de novo* synthesis, and only a small portion is supplemented by feeding, which indicates that yolk cholesterol might be affected by both genetic and nutritional factors (Griffin, 1992; Klkin et al., 1997). Previous studies have reported that the yolk cholesterol concentration varied among breeds ranging from 10 to 100 mmol/L with a normal distribution and was positively correlated with embryo mortality during hatching (Panda et al., 2003; Yang et al., 2013). These observations support the notion that genetic factors might regulate yolk cholesterol. Moreover, cholesterol is found at relatively low levels in feeding, which further suggests that yolk cholesterol is mainly affected by the genetic makeup of the bird (Sreenivas et al., 2013). Accordingly, if the heritability of yolk cholesterol is high, then individual selection could be used. However, if the heritability of yolk cholesterol is moderate or low, then sire selection should be preferred.

In mice, oocyte-derived bone morphogenetic protein 15 (BMP15) and growth differentiation factor 9 (GDF9) have been shown to promote cholesterol biosynthesis in cumulus cells as a compensation mechanism for cholesterol production deficiencies in the oocyte (Su et al., 2008). Furthermore, the *cyp19a1*, *cyp17a1*, tesc, *apoc1*, and *star* genes have been reported to play roles in the regulation of steroidogenesis during oocyte maturation in both trout and Xenopus (Gohin et al., 2010). Moreover, feeding hens with a diet supplemented in alfalfa saponin extract has been shown to decrease the yolk cholesterol content. This decrease in yolk cholesterol was associated with increased expression levels of cholesterol 7 alpha-hydroxylase and apolipoprotein H in the liver and decreased expression levels of very low-density lipoprotein (VLDL) receptor, apolipoprotein B, apovitellenin-1, and vitellogenin in the oocyte (Zhou et al., 2014). Nevertheless, little remains known about the molecular mechanisms underlying the regulation of yolk cholesterol in chicken.

In this study, we used as an experimental model a group of Wenchang chickens, an indigenous Chinese breed with a detailed pedigree. Egg quality was determined in two consecutive generations, and genetic parameters were evaluated in individuals and sire families. Moreover, follicular membrane was collected from hens with either low or high yolk cholesterol content, and transcriptional sequencing was used to screen for candidate genes and signal pathways involved in the regulation of cholesterol synthesis.

## Materials and Methods

### Birds Management

All birds used in this study were Wenchang chickens, a Chinese indigenous breed with a complete pedigree. A total of 24 sire families (24 males and 240 females) and 362 daughters (equality distributed among the sire families with pure breeding) were raised with one bird *per* cage and maintained on a 16 L/8 D (16 h light and 8 h dark) photoperiod during egg laying. At 40 weeks of age, eggs and follicular tissues were collected for quality and yolk cholesterol analysis. Hens were artificially inseminated, and all birds were kept at 15–20°C during the egg-laying period. Egg quality and cholesterol content were determined in two successive generations.

All experimental procedures were performed following guidelines developed by the China Council on Animal Care and Protocols and were approved by the Animal Care and Use Committee of Anhui Agricultural University, China (permission no. SYDW-P2017062801).

### Egg Quality and Yolk Cholesterol Analysis

Three eggs were collected from each bird within five consecutive days, and egg quality was assessed within 24 h after collection. A digital scale (accuracy: 0.01 g) was used to measure the weight of each egg. An electronic digital caliper was used to measure the longitudinal diameter (LE) and the transverse diameter (WE) of each egg, and the egg shape index was defined as the WE/LE ratio. Shell strength was measured using an eggshell force gauge (model II, Robotmation, Tokyo, Japan). Then, the egg was broken onto a flat surface, and the height of the inner thick albumen (egg white) was measured using an egg analyzer (model EA-01, ORKA Food Technology, Ramat HaSharon, Israel). The yolk was separated from the albumen, weighed, and stored at -20°C for cholesterol determination. The shell thickness was measured using a digital Vernier caliper (model NFN380, Fujihira Industry, Tokyo, Japan).

After weighing the yolk, ∼0.1 g of yolk was transferred to a 1.5-ml tube. Nine times by weight of anhydrous ethanol were added to the yolk, and the mixture was mechanically homogenized for 30 s at 50 Hz in an ice water bath. Next, all samples were centrifuged for 10 min at 2,500 rpm, and 25 µl of the supernatant was transferred into a well of a 96-well plate. After adding 250 μl of working solution (50 mmol/L Good’s buffer, 5 mmol/L phenol, 0.3 mmol/L 4-AAP, ≥50 KU/L cholesteryl esterase, ≥25 KU/L cholesterol oxidase, and ≥1.3 KU/L peroxidase) to each well, the solution was mixed and incubated for 10 min. The optical density (OD) was measured at wavelength of 510 nm, and the cholesterol content was calculated using the following formula: cholesterol content (mg) = (sample OD - blank OD)/(corrected OD - blank OD) × dilution factor × yolk weight × 386.6535/1,000.

### Follicular Tissue Collection, Total RNA Extraction, and cDNA Library Construction

After yolk cholesterol been determined, birds with the lowest (L group) and highest (H group) yolk cholesterol content were selected for follicular tissue collection. For each group, three hens at 41 weeks of age were killed ∼22 h after ovulation, and then, the ovaries were collected rapidly and kept on ice. Three largest (25–30 mm) yellow preovulatory follicles were isolated from each ovary. The yolk was squeezed out, and the granulosa layer was collected, divided into two parts, and immediately stored in liquid nitrogen for RNA isolation.

Total RNA was isolated from individual samples using the OMEGA total RNA extraction kit (Omega Bio-Tek, Norcross, GA, USA) according to the manufacturer’s recommendations. RNA integrity number and quality were analyzed using an Agilent 2100 Bioanalyzer (Agilent Technologies, Santa Clara, CA, US). Then, qualified total RNA was further purified using an RNase-Free DNase Set (Qiagen, Hilden, Germany). Purified total RNA was used for the construction of a complementary DNA (cDNA) library and subsequent sequencing (NEB Next Ultra Directional RNA Library Prep Kit for Illumina; New England Biolabs, Ipswich, MA, USA). The remaining RNA from each sample was reverse transcribed and stored at -80°C for RNA sequencing (RNA-Seq) results validation *via* real-time quantitative PCR (RT-qPCR).

### RNA-Seq

Following messenger RNA purification using Agencourt AMPure XP beads (Beckman, Brea, CA, USA), the first and second cDNA strands were synthesized using the SuperScriptII Reverse Transcriptase (Invitrogen, Carlsbad, CA, USA) according to the manufacturer’s recommendations. Next, double-stranded cDNA was end repaired, adenylated, and ligated to NEBNext Adaptors (New England Biolabs) according to the manufacturer’s recommendations. The cDNA fragments of 150–200 bp were selected using the Agencourt AMPure XP system (Beckman), and PCR was performed using the Phusion High-Fidelity DNA polymerase (New England Biolabs), universal PCR primers, and an Index (X) primer. Clustering of the index-coded samples was performed on a cBot Cluster Generation System using the TruSeq PE Cluster Kit v3-cBot-HS (Illumina, San Diego, CA, USA) according to the manufacturer’s recommendations. After clustering, the libraries were sequenced using a paired-end 2 × 125 bp lane on an Illumina HiSeq 4000 platform (Shanghai Personal Biotechnology, Shanghai, China).

### Filtering of Raw Data and Mapping of High-Quality Reads to the Chicken Reference Genome

Six libraries from each group (*n* = 3) were sequenced. First, raw reads in FASTQ format were filtered to generate clean reads by removing reads containing adapters or ambiguous nucleotides and reads of low quality, as described by Wang et al. (2017a). Then, the filtered reads were mapped to the chicken reference genome (Gallus_gallus-5.0) using the spliced mapping algorithm of Tophat (version 2.0.9) with no more than two mismatches. Basic mapping statistics, mapped reads distribution across the chicken genome, and annotated genes were determined to evaluate the randomness of the distribution.

### Calculation of Gene Expression Level

Gene expression level was calculated using the Cufflinks suite (version 2.1.1) on Tophat output. In brief, the specific gene location was obtained using gene annotation, and the number of reads covering this location was counted. Then, the gene expression level was normalized using the following formula: fragments *per* kilobase million (FPKM) = transcription reads/(transcription length × total mapped reads in the run) × 10^9^.

### Differentially Expressed Genes Analysis

The normalized FPKM values were used as gene expression levels for the analysis of differentially expressed genes (DEGs) using the Cuffdiff program of the Cufflinks suite (v2.1.1). The differences in gene expression were evaluated using the fold change (≥2.0) and Fisher’s exact test (false discovery rate ≤ 0.05).

### Functional Annotation of DEGs

For the analysis of Gene Ontology (GO) term enrichment, the DEGs were first annotated with GO terms, and the number of DEGs for each GO term was calculated. Then, the hypergeometric test was used to identify GO terms that were significantly enriched in DEGs when compared to the chicken reference genome. The enrichment was calculated using the following formula: enrichment = (*m*/*n*)/(*M*/*N*), where *N* is the total number of genes annotated with a GO term, *n* is the number of DEGs in *N*, *M* is the total number of genes annotated with a specific GO term, and *m* is the number of DEGs in *M*. The *p* values were then adjusted by applying the Bonferroni correction, and a *p* value of 0.05 was set as the threshold for adjusted *p* values (false discovery rate). A similar method was used for the analysis of the Kyoto Encyclopedia of Genes and Genomes (KEGG) pathway enrichment, except genes were assigned to KEGG pathways instead of being annotated with GO terms.

### RT-qPCR Verification of the RNA-Seq Data

RT-qPCR was performed to validate the RNA-Seq results, using the TB Green Premix Ex Taq (Takara, Shiga, Japan) with SYBR Green Dye and the same RNA samples that were used for RNA-Seq. Seven genes were selected for RT-qPCR verification. The primers used for these genes are listed in **Table 1**. The reactions were performed in a total volume of 20 μl according to the manufacturer’s recommendations, using an ABI PRISM 7500 sequence detection system (Applied Biosystems, Madrid, Spain) and the following conditions: 5 min at 94°C (1 cycle); 30 s at 94°C, 30 s at annealing temperature (according to the primers listed in **Table 1**), and 30 s at 60°C (35 cycles); and melting curve from 55 to 94°C. Glyceraldehyde 3-phosphate dehydrogenase was selected as the endogenous reference gene, and genes from the L group were set as the criterion. The expression levels were calculated using the 2^-ΔΔCT^ method.

**Table 1:** Primers used for RT-qPCR verification of the RNA-Seq data.

No.	Gene symbol	Ensembl accession no.	Primer sequence (5′–3′)	Annealing temperature (°C)
1	CCL19	ENSGALG00000028256	GAAGCTTTAGGGGGAGCCAATCCTCTAAGACCTCTCCGGG	57
2	OSMR	ENSGALG00000003747	TAACTAAAGCAGCGGAGTGCTTTCCCGGGGAGGGTTATCA	55
3	ALOX5	ENSGALG00000005857	CAAACACACGGGAAACCACCCCACCGTCACATCGTAGGAG	57
4	FABP3	ENSGALG00000037050	CCTGGAAGCTGGTGGATACGCCGTGGTCTCATCGAACTCC	59
5	ApoA1	ENSGALG00000007114	GGACCGCATTCGGGATATGGACTTGGCGGAGAACTGGTC	57
6	CYP19A	ENSGALG00000013294	ATGGGGATTGGAAGTGCCTGTCATGAAGAAAGGGCGGACC	57
7	LPL	ENSGALG00000015425	CCCACTGAAACTTTTTCGCCGCTGTCCAGGAACCAGGTAGC	57

### Statistical Analysis

All statistical analyses were performed using the SAS 9.3 software (SAS, Cary, NC, USA). Heritability was analyzed using the half-sib correlation method and evaluated using the VARCOMP procedure with the restricted maximum likelihood option. Differences in egg quality among individuals and sire families were compared using the ANOVA procedure. Differences in egg quality between the two consecutive generations were compared using the independent *t* test procedure. The univariate procedure was used to test the normal distribution of the yolk cholesterol content. The general linear model procedure least squares linear model was used to analyze the phenotypic correlation between yolk cholesterol content and egg quality among female individuals and sire families. All data were expressed as mean values ± standard deviation (SD).

## Results

### Egg Quality and Its Correlation With Yolk Cholesterol Content

Among first-generation female individuals, the egg weight, egg shape index, shell strength, shell thickness, yolk weight, egg white height, Haugh unit, and cholesterol content were 45.36 g, 0.81, 3.07 kg/cm^2^, 0.340 mm, 15.57 g, 3.36 mm, 58.70, and 45.86 mmol/L, respectively. Among second-generation female individuals, the egg weight, egg shape index, shell strength, shell thickness, yolk weight, egg white height, Haugh unit, and cholesterol content were 45.16 g, 0.80, 2.97 kg/cm^2^, 0.338 mm, 15.57 g, 3.32 mm, 58.42, and 45.25 mmol/L, respectively (**Table 2**). Accordingly, none of the indexes assessed differed significantly between the two generations.

**Table 2:** Egg quality among first- and second-generation female individuals and sire families.

Source	Generation	Egg weight (g)	Egg shape index	Shell strength (kg/cm^2^)	Shell thickness (mm)	Yolk weight (g)	Egg white height (mm)	Haugh unit	Cholesterol (mg/egg)
Females	1	45.36 ± 4.44	0.81 ± 0.12	3.07 ± 0.92	0.340 ± 0.032	15.57 ± 1.64	3.36 ± 1.15	58.70 ± 12.33	274.3 ± 36.73
	2	45.16 ± 4.02	0.80 ± 0.08	2.97 ± 0.84	0.338 ± 0.031	15.57 ± 1.57	3.32 ± 0.86	58.42 ± 8.90	265.2 ± 22.88
Sire families	1	44.81 ± 2.89	0.82 ± 0.06	3.07 ± 0.51	0.337 ± 1.72	15.52 ± 0.77	3.36 ± 0.26	58.86 ± 3.08	285.2 ± 128.1
	2	43.88 ± 1.87	0.78 ± 0.02	3.83 ± 0.403	0.373 ± 0.016	13.72 ± 0.61	4.68 ± 0.301	60.20 ± 3.52	282.7 ± 53.5

Phenotypic correlation analyses (**Table 3**) suggested that a higher egg weight was associated with an increase in yolk weight, shell strength, shell thickness, and egg white height (*p* < 0.05), and a decrease in the egg shape index (*p* < 0.05). While higher yolk cholesterol was not associated with changes in egg quality among female individuals, higher yolk cholesterol was, however, associated with an increase in yolk weight, egg white height, and yolk color in sire families (*p* < 0.05) of Wenchang chicken.

**Table 3:** Correlation between the level of cholesterol in egg yolk and egg quality indexes.

Source	Trait	Egg weight	Yolk weight	Egg shape index	Shell thickness	Shell strength	Egg white height	Haugh unit
Females	Cholesterol	0.573	0.978	0.412	0.152	0.432	0.155	0.142
	Egg weight		<0.001	<0.001	<0.001	0.607	0.520	0.101
	Yolk weight			<0.001	<0.001	0.082	0.042	0.238
Sire families	Cholesterol	0.375	<0.001	0.118	<0.001	0.387	<0.001	0.341
	Egg weight		<0.001	<0.001	<0.001	0.006	0.010	0.653
	Yolk weight			<0.001	<0.001	0.747	0.405	0.572

### Heritability Evaluation

Among female individuals, the heritability estimates for egg weight, egg shape index, shell strength, shell thickness, yolk weight, and cholesterol content were 0.432, 0.024, 0.030, 0.374, 0.146, and 0.328, respectively (**Table 4**). Among sire families, the heritability estimates for egg weight, egg shape index, shell strength, shell thickness, yolk weight, and cholesterol content were 0.354, 0.070, 0.206, 0.516, 0.176, and 0.530, respectively (**Table 4**). Accordingly, the evaluation of egg weight, shell thickness, and cholesterol content resulted in high heritability estimates for each parameter, while the evaluation of yolk weight and egg shape index resulted in medium and low heritability estimates, respectively. Furthermore, the evaluation of shell strength, shell thickness, and cholesterol content in sire families resulted in higher heritability estimates for each parameter.

**Table 4:** Paternal half-sib family structure and heritability estimates.

Trait	Egg weight (g)	Egg shape index	Shell strength (kg/cm^2^)	Shell thickness (mm)	Yolk weight (g)	Egg white height (mm)	Haugh unit	Cholesterol (mg/egg)
Sires	24	24	24	24	24	24	24	24
*K*	7.27	7.27	7.27	7.27	7.27	7.27	7.27	7.27
Progeny	362	362	362	362	362	362	362	362
Heritability								
Females	0.432	0.024	0.030	0.374	0.146	/	/	0.328
Sire families	0.354	0.070	0.206	0.516	0.176	/	/	0.530

K = (N - Σn_i_2/N)/(S - 1), N = total number of progeny, n_i_ = number of progeny for sire i, and S = number of sires.

### RNA-Seq Data and Transcriptome Assembly Results

The sequenced libraries generated an average of 42,290,686 ± 870,109 raw reads *per* library. After filtering using the Q20 standard, the average number of clean reads *per* library was 41,785,132 ± 943,074 with a clean read ratio of 98.80 ± 0.34%. Among the filtered clean reads, an average of 35,157,925 ± 900,332 reads *per* library was mapped to the chicken reference genome with a mapping ratio of 84.14 ± 0.97%. Finally, an average of 28,863,072 ± 981,091 reads *per* library was mapped to genes with a mapping ratio of 85.39 ± 2.25%. The clean reads mapped mostly to gene exons with a ratio of 97.56 ± 0.34%, and according to the sequencing results, an average of 15,613 genes was mapped (**Supplementary Table 1**).

### Identification of Candidate Genes Involved in Cholesterol Metabolism

#### GO Term Analysis of the DEGs

**Figure 1:**
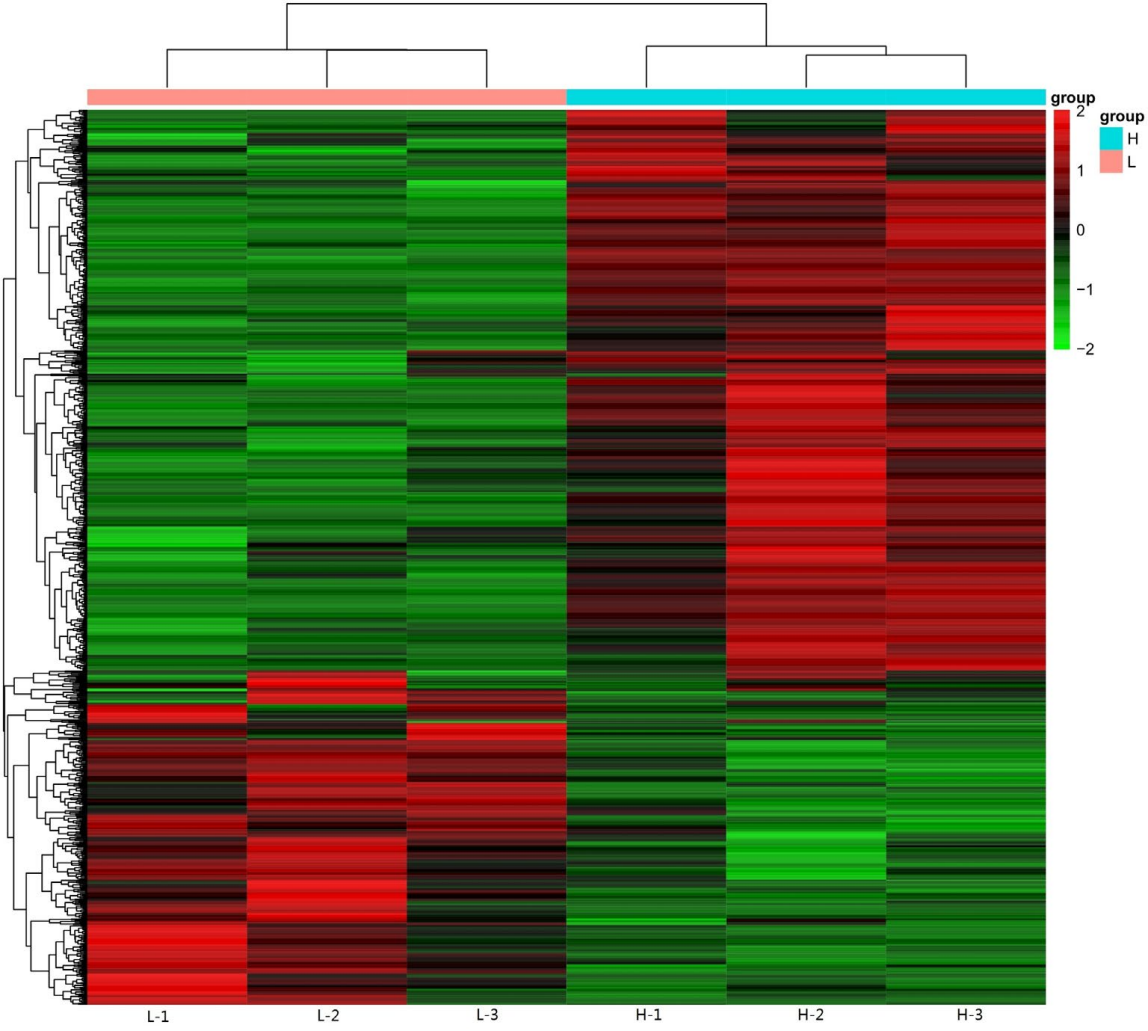
Heatmap analysis of key genes involved in yolk cholesterol deposition. Each row represents a single gene, and each column corresponds to a sequenced sample. The level of expression of each gene is color coded with green and red representing low and high expression levels, respectively.

The data from two groups, chickens with the highest and lowest levels of yolk cholesterol, were compared to identify genes with differing reads *per* kilobase *per* million values. Compared to chickens with the lowest level of yolk cholesterol, a total of 375 and 578 genes were down- and upregulated, respectively, in chickens with the highest level of yolk cholesterol (**Figure 1** and **Supplementary Table 2**).

**Figure 2:**
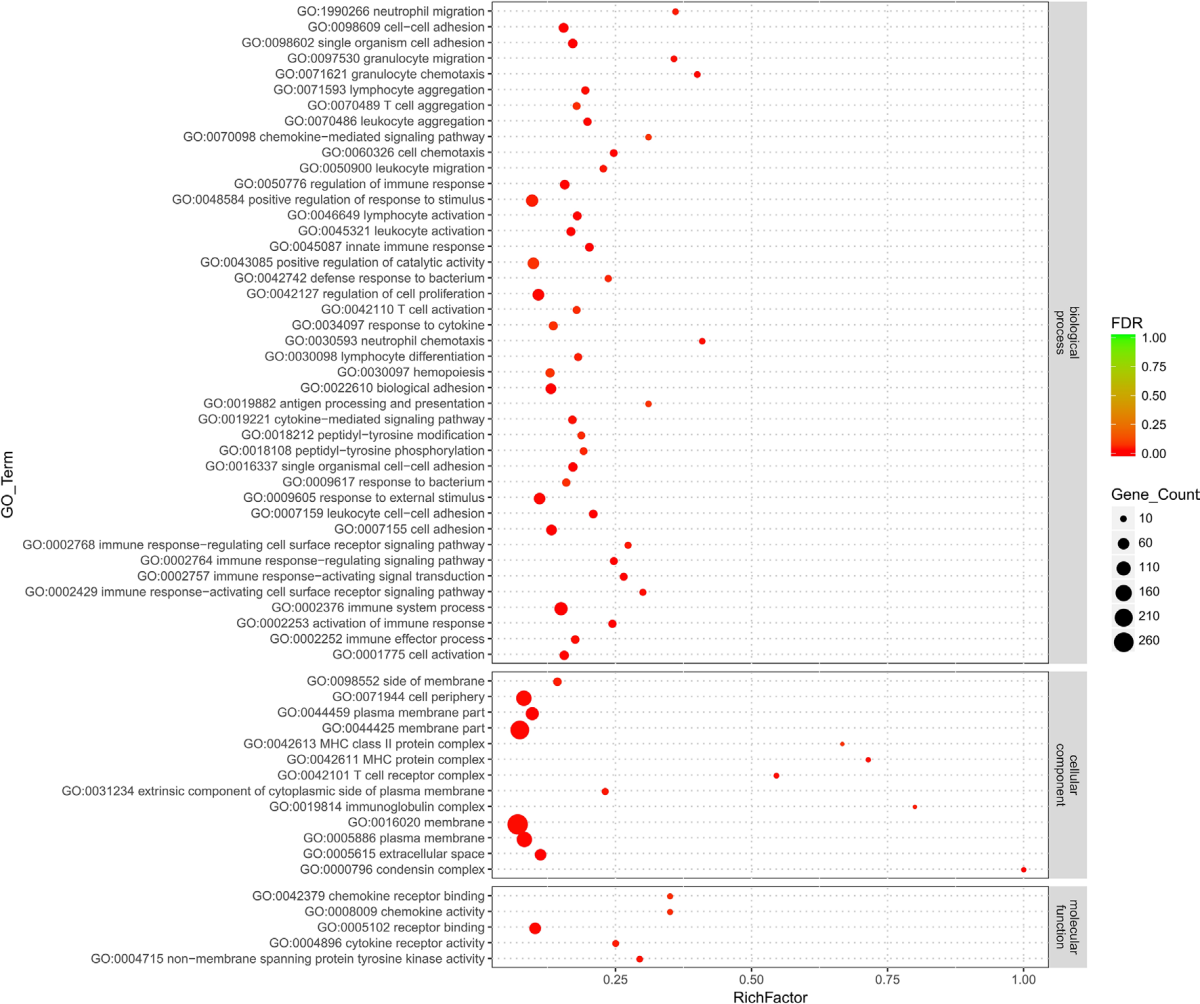
Gene Ontology (GO) term enrichment analysis of candidate genes. The scatter plot presents the results of the GO term enrichment analysis for the candidate genes. The *y*-axis shows the GO terms significantly enriched (*p* < 0.05), and the *x*-axis shows the log 10 *p* values. The size of the bubble corresponding to a specific GO term indicates the number of candidate genes annotated with this term.

All the DEGs were subjected to GO term and KEGG pathway enrichment analyses. In total, 559 genes were assigned to 2,251 biological processes, 316 cellular components, and 434 molecular functions (**Supplementary Table 3**). Out of these, 42 biological processes, 13 cellular components, and 5 molecular functions were significantly enriched (*p* < 0.05) (**Figure 2**).

Among the various biological processes assigned, positive regulation of response to stimulus (GO:0048584) is the largest category with a total of 749 genes included, and ∼13.36% (72 out of 539) of the candidate genes were annotated with this term. Furthermore, two categories of GO terms associated with biological processes were highly represented: GO terms related to cell–cell adhesion (9 GO terms) and the immune response (25 GO terms). Out of these, the GO terms immune system process (GO:0002376) and immune response (GO:0006955) were significantly enriched (**Supplementary Table 3** and **Supplementary Figure 1**). Moreover, the *MYO1G*, *B2M*, *CCL19*, and *CD79B* highly enriched genes were annotated with more than three biological process categories related to the immune response, while the *LCK*, *VAV3*, and *CCLi8* highly enriched genes were annotated with the cell adhesion biological process category (**Supplementary Table 3**).

Regarding cellular component categories, membrane (GO:0016020) and membrane part (GO:0044425) were the two most represented GO terms with 4,104 and 3,114 genes included, respectively. Out of 559 candidate genes, 284 and 228 were assigned to the membrane and membrane part categories, respectively. Furthermore, genes annotated with the GO term condensin complex (GO:0000796) were highly enriched (**Supplementary Figure 2**), and remarkably, all the genes annotated with this GO term were downregulated. Considering the role of transport or secretion through the follicle membrane in cholesterol formation, membrane functions are of particular interest. Among the 13 significantly enriched GO terms for cellular components, 9 are related to the membrane, and 17 enriched genes, including *B2M*, *ALOX5*, *LCP1*, and *LPL*, were annotated with more than 3 membrane-related GO terms.

Lastly, five molecular function categories were enriched (**Supplementary Table 3**), and notably, all the genes annotated with the GO term nonmembrane spanning protein tyrosine kinase activity (GO:0004715) were upregulated. Furthermore, the *CCL4*, *CCL5*, and *CCL19* highly enriched genes were annotated with the signal transport GO term and were all upregulated (**Supplementary Figure 3**).

#### KEGG Pathway Analysis of the DEGs

**Figure 3:**
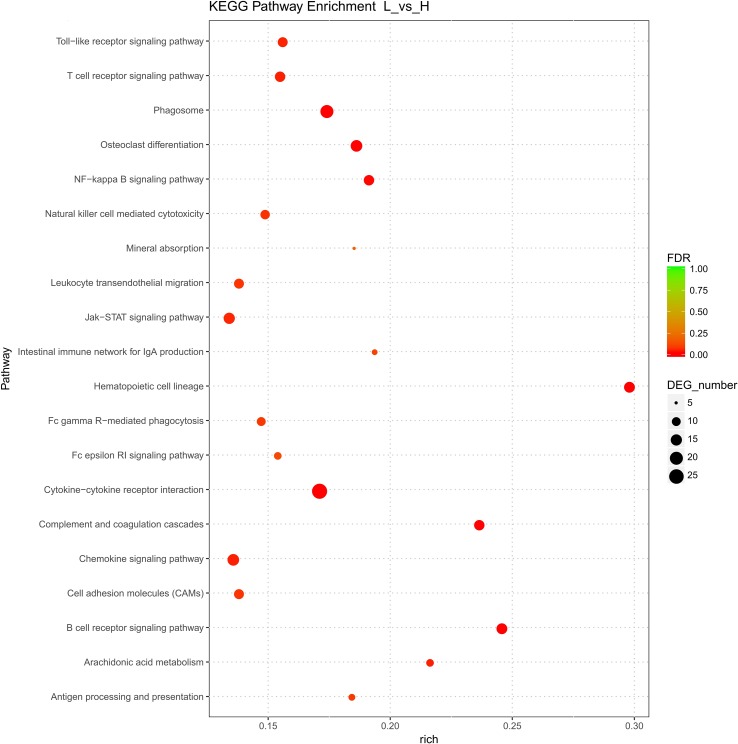
Kyoto Encyclopedia of Genes and Genomes (KEGG) pathway analysis of differentially expressed genes. The x-axis shows the enrichment score, the size of a bubble indicates the log value of the number of genes enriched in a pathway, and the color shade represents the *p* value determined using Fisher’s exact test.

In total, 27 KEGG pathways were significantly enriched (*p* < 0.05). They involved 151 genes, 123, and 28 of which were up- and downregulated, respectively. Among the significantly enriched pathways, three were related to signaling interactions and cell transport, and each one of these three pathways involved more than 20 DEGs (**Figure 3** and **Supplementary Table 4**). Furthermore, the highly enriched KEGG pathways were mainly associated with signal transduction, lipid metabolism, and the endocrine system (**Figure 3**). Notably, hematopoietic cell lineage was the most significantly enriched KEGG pathway for the DEGs highly expressed in follicles with the highest level of cholesterol. Moreover, the arachidonic acid metabolism, mineral absorption, PI3K-Akt signaling, ovarian steroidogenesis, and peroxisome proliferator-activated receptors (PPARs) signaling KEGG pathways were involved in the development of follicles with different cholesterol contents. Six genes were involved in ovarian steroidogenesis, among which *CYP2J*, prostaglandin-endoperoxide synthase 2 (*PTGS2*), *ALOX5*, and *ADCY7* were upregulated, while *CYP19A1* and phospholipase A2 group IVF (*PLA2G4F*) were downregulated. Interestingly, *ALOX5* was also annotated with two GO terms (extracellular space and membrane).

### Expression of DEGs Involved in the Development of Follicles With Different Cholesterol Contents

We found that many DEGs were involved in the development of follicles with different cholesterol contents, including *B2M*, *ALOX5*, *LCP1*, *LPL*, *FABP3*, *APOA1*, *FLRT2*, *GPRC5B*, *GOLM1*, *GLDN*, and others. Within this list, 23 genes were mapped to the sex chromosome Z, including *LPL*, *CCL19*, *OSMR*, *GOLM1*, and *SYK*.

**Figure 4:**
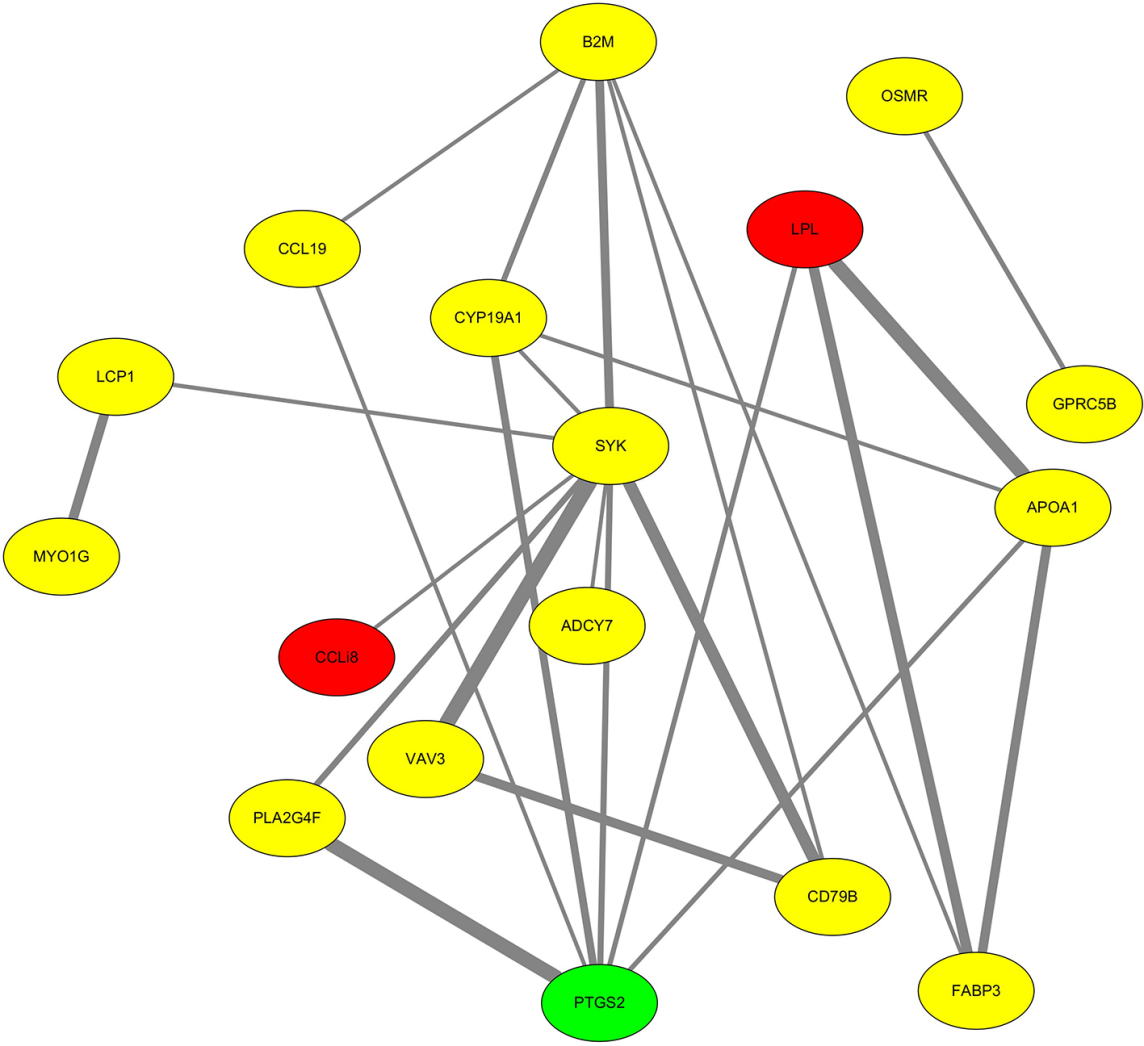
Protein–protein interaction network analysis of selected differentially expressed genes. Proteins highlighted in red and green were significantly down- and upregulated, respectively, while proteins highlighted in yellow showed no significant difference. In the network, each line represents the strength of the relationship between two proteins. Strong interactions are indicated by high STRING combined scores and wide lines, while weak interactions are indicated by low STRING combined scores and narrow lines.

Next, the highly enriched DEGs were mapped to the chicken protein–protein interaction networks of the STRING database (https://string-db.org). The Cytoscape software was then used to produce a protein–protein interaction plot (**Figure 4**). Lipoprotein lipase (LPL) was significantly downregulated in follicular cells with the highest level of cholesterol and had strong protein–protein interactions, as reflected by high STRING combined scores (the combined score is based on the evidence in the STRING database and reflects the level of confidence of a protein–protein interaction). Meanwhile, *PTGS2* was upregulated and exhibited strong protein–protein interactions (i.e., high STRING combined scores).

**Figure 5:**
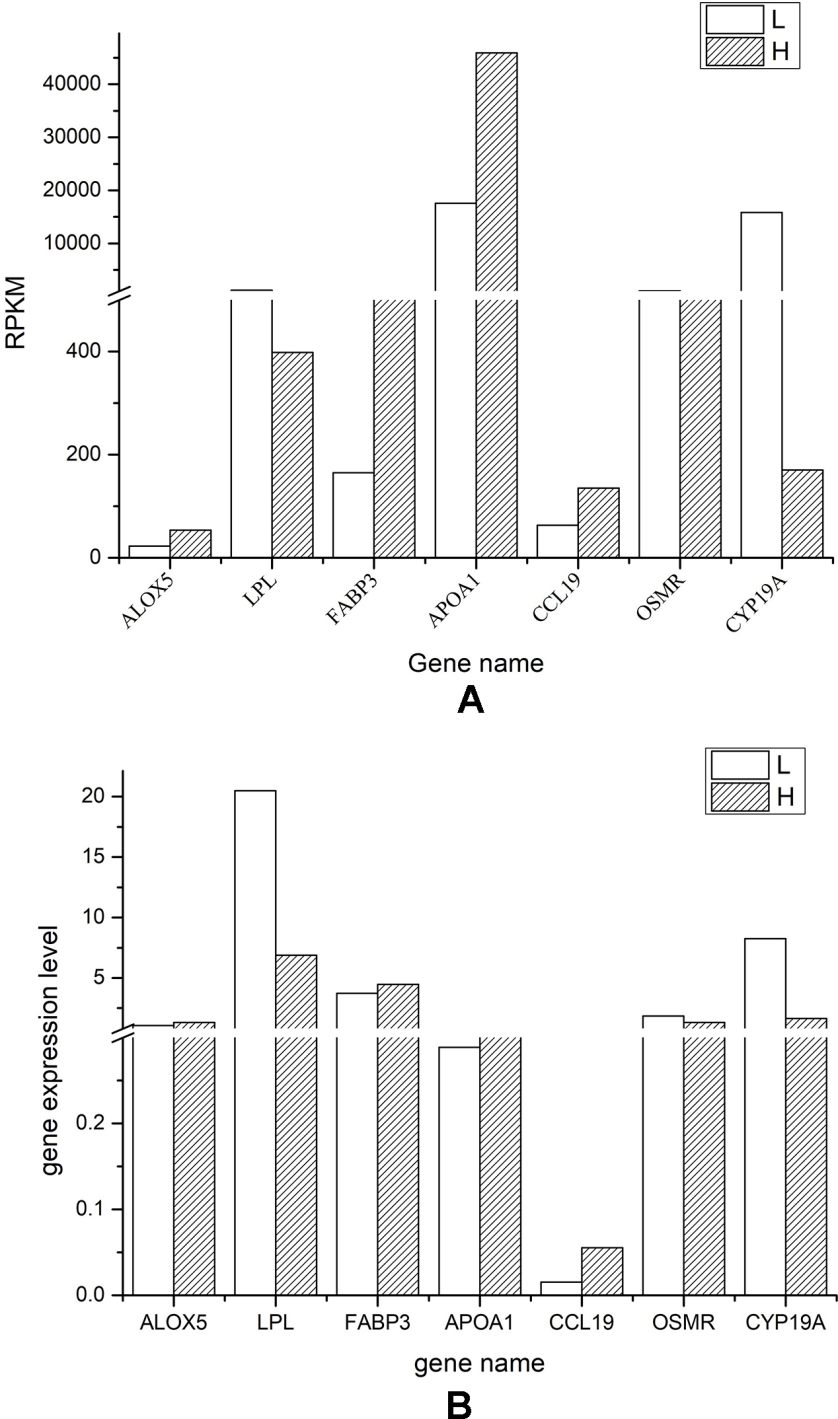
Validation of the RNA-Seq results *via* real-time quantitative PCR RT-qPCR analyses. **(A)** Diagram showing the reads *per* kilobase *per* million value of each gene in both low (L) and high (H) cholesterol content groups. **(B)** Diagram showing the expression level quantified by RT-qPCR of the indicated genes in both L and H groups.

We selected seven DEGs (both up- or downregulated in chicken follicular cells with the highest level of cholesterol) and compared the messenger RNA quantification from the transcriptional sequencing results with the expression level assessed by RT-qPCR. Globally, we found a good correlation for the expression trend of the selected genes, as measured by RNA-Seq and RT-qPCR (**Figure 5**). However, the expression of *ALOX5* and *OSMR* exhibited no difference between the H and L groups when measured by RT-qPCR. Furthermore, the detected expression level of *CCL19* was relatively low, while the expression level of *LPL* and *CYP19A* was significantly higher in follicular cells from the L group than in that from the H group.

## Discussion

In agreement with a previous report by Baumgartner et al. (2008), this study did not find evidence of a significant association between the yolk cholesterol content and various indexes of egg quality. Accordingly, these observations suggested that the yolk cholesterol content could not be regarded as a standard index for egg quality. Furthermore, Ledur et al. (2000) reported that egg quality differed among individuals and increased with age, which suggested that layer performance might be improved by performing selection at an older age. Moreover, the male line is expected to improve egg production at the end of the cycle (Bulut et al., 2013; Goraga et al., 2013). Therefore, it has been proposed that cholesterol synthesis might be affected by the sire family and could be regulated by genes located on chromosome Z (Ledur et al., 2000). Our analyses suggested that the egg weight, shell strength, shell thickness, and egg shape index were correlated with the yolk weight. Indeed, a heavier egg yolk might require more surrounding egg white and shell, which would result in higher egg weight. While the weight of the egg yolk depended on follicular development, the cholesterol content of the egg yolk was positively correlated with the egg weight, which suggested that cholesterol and egg yolk were the most important factors affecting egg weight (Baumgartner et al., 2008). In general, a relatively high cholesterol content has been associated with good health conditions in birds, whereas higher nutrient content in the egg yolk has been associated with a higher egg weight (Zhang, 2016).

In this study, the heritability estimate for the egg weight in Wenchang chickens was 0.432 in females and 0.354 in sire families. Overall, these estimates are in agreement with a previous study by Rath et al. (2015), which reported a heritability estimate of 0.443 for the egg weight in white leghorns chickens. Furthermore, in this study, shell thickness was positively correlated with shell weight, and the estimated heritability of shell strength (0.030 in females and 0.206 in sire families) was consistent with previous reports (Rath et al., 2015; Alwell et al., 2018). For the moderate heritability of shell strength in sire family, it might be more appropriate for sire selection to achieve a quick progress in breeding. In contrast, the egg shape index and yolk weight had relatively low heritability estimates, which might be due to the high phenotypic variance, and further suggested that these two traits could not be selected using phenotypic values. Lastly, the heritability estimate for yolk cholesterol content was higher in sire families than in females, which further indicated that yolk cholesterol content was controlled by genes located on the chromosome Z and could be selected through the male line (Ledur et al., 2000).

Ovarian follicle development requires markedly increased DNA and protein synthesis in the granulosa cells of the follicle membrane (Seol et al., 2006; Bonnet et al., 2011). During the rapid growth of chicken follicles, DNA and protein synthesis is stimulated and regulated by a variety of steroid hormones (Diaz, 2011) and the expression of genes involved in this progress. For example, the biological function of the phospholipase A2 (PLA2) subfamily of enzymes is to catalyze the hydrolysis of the sn-2 position of membrane glycerophospholipids, which leads to the production of free fatty acids and lysophospholipids (Duncan et al., 2008). Furthermore, several reports have involved PLA2 in the induction of cell apoptosis. In chickens with the highest yolk cholesterol content, the downregulation of *PLA2G4* in the ovarian steroidogenesis pathway suggested that increased phospholipids synthesis was required for cholesterol deposition (Diouf et al., 2006; Aljakna et al., 2012). Moreover, PTGS2 has been reported to be induced or upregulated by the luteinizing hormone surge during ovulation in rodent and fish (Yerushalmi et al., 2014; Tang et al., 2017). Therefore, the upregulation of *PTGS2* in follicles might also suggest that ovulation occurs more frequently in chickens producing eggs with a higher cholesterol content. Indeed, the increased level of PTGS2, together with the action of arachidonate-5-lipoxygenase (ALOX5), would further promote the release of arachidonic acid (Kurusu et al., 2009), and the subsequent conversion of arachidonic acid by downstream metabolic enzymes of the CYP2J subfamily could impact the ovulatory mechanisms (Newman et al., 2004).

The expression of LPL in the ovarian follicles of domestic chicken was first identified by Benson et al. (1975). LPL is an essential enzyme of VLDL metabolism and exhibits high levels of expression in rapidly growing ovarian follicles, which provides follicular tissues with the enzyme required to hydrolyze VLDL into fatty acids and monoglycerides (Gupta et al., 2017). In the present study, LPL was expressed at a relatively low level in the ovarian follicles with the highest cholesterol content. Therefore, we would like to propose that low levels of LPL play a role in the retention of high VLDL levels, which in turn leads to an increase in the amount of VLDL-cholesterol and triglyceride-rich lipoproteins in ovarian follicles. Furthermore, VLDL has been demonstrated to be a source of neutral lipids in the oocytes of anguillid eels and cutthroat trouts (Damsteegt et al., 2015; Lubzens et al., 2017). Moreover, the downregulation of *LPL* has been involved in the PPAR signaling pathway. As part of the PPAR signaling pathway, ApoA1 and FABP3 play roles in lipid metabolism (Wang et al., 2017b), while PEPCK plays a role in gluconeogenesis (Glorian et al., 2001). ApoA1, FABP3, and PEPCK are also all upregulated in response to retinoid X receptor alpha. Unlike mammalians where females have XX and males XY sex chromosomes, birds have the ZW system where females have ZW and males ZZ sex chromosomes. In male chickens, it has been shown that the two copies of chromosome Z are not affected by global dosage compensation mechanisms, and therefore, genes located on chromosome Z usually exhibit higher levels of expression in males than in females (Toups et al., 2011). The *LPL* gene is assigned to chromosome Z and usually exhibits a low level of expression in birds (Han, 2005), which might explain the negative correlation with the yolk cholesterol content in sire families.

In mammals, pregnancy will improve the innate and adaptive immunity during gestation to increase pregnancy outcomes (Kraus et al., 2012). Similar to mammals’ pregnancy, follicles formation and ovulation in chickens may also need improved immunity to guarantee a higher egg quality. During the rapid growth phase of ovarian follicles, the components of the follicle matrix expand rapidly, which acts as intrinsic mechanical stress during the accumulation of yolk precursors (Kraus et al., 2012; Richards et al., 2008). We speculated that, in the follicles with the highest cholesterol content, this phenomenon was responsible for the increased expression of genes related to the immune response and signaling pathways, including hematopoietic cell lineage, toll-like receptor signaling pathway, and others.

Energy and substrate sources are also required for ovarian folliculogenesis (Seol et al., 2006). Interestingly, genes related to the arachidonic acid metabolism, which contributes to energy intake, were significantly enriched in ovarian follicles with a high cholesterol content (Lee et al., 2005). Our data suggested that VLDL absorption as a yolk precursor in ovarian follicles with the highest cholesterol content was mediated through the downregulation of *LPL* expression. This contrast with the situation in mammals, where phospholipase A2 group IVA (*PLA2G4A*) expression is upregulated in granulosa cells at ovulation (Diouf et al., 2006), and the yolk exhibits a higher content of arachidonic acid through the down- and upregulation of *PLA2G4F* and *PTGS2*, respectively. Furthermore, in cows, the upregulation of *PLA2G4A* has been associated with a down- and upregulation of *CYP19A1* and *PTGS2*, respectively (Sirois, 1994). These differences might indicate that a high cholesterol content requires arachidonic acid degradation and *PLA2* downregulation to maintain high levels of phospholipids while keeping the same expression trend for *CYP19A1* and *PTGS2*.

## Conclusions

The yolk cholesterol content was most affected by the sire family with a heritability estimate of 0.530. Furthermore, the ovarian steroidogenesis pathway appeared to affect the yolk cholesterol content, with the downregulation of the *LPL* gene located on chromosome Z playing key roles. In contrast to mammals, a high yolk cholesterol content appeared to require the downregulation of *PLA2G4A* in chickens, which might also affect ovulation. Nevertheless, further studies with *LPL* overexpression or knockdown are required to confirm its role in the functional regulation of the yolk cholesterol content in birds.

## Data Availability Statement

The data used in this manuscript can be found according to the link below: https://www.ncbi.nlm.nih.gov//bioproject/PRJNA532290.

## Ethics Statement

All experimental procedures were performed following guidelines developed by the China Council on Animal Care and Protocols and were approved by the Animal Care and Use Committee of Anhui Agricultural University, China (permission No. SYDW-P2017062801).

## Author Contributions

XC designed the study, analyzed and interpreted the data, and wrote the paper. WZ conducted egg quality measurement and follicle membrane collection. YD conducted qPCR experiments. XL extracted RNA from follicle membrane. ZG designed the study.

## Funding

Support for this project was provided in part by the Major Scientific and Technological Special Project in Anhui Province (18030701174), the Open Fund of Anhui Province Key Laboratory of Local Livestock and Poultry, Genetical Resource Conservation and Breeding (AKLGRCB2017001), and the Key project of natural fund of Anhui Provincial Education Department (KJ2018A951).

## Conflict of Interest

The authors declare that the research was conducted in the absence of any commercial or financial relationships that could be construed as a potential conflict of interest.

## Supplementary Material

Supplementary Figure 1 Directed acyclic graph (DAG)display of GO highly enriched biological process results with candidate targeted genes. The enrichment of GO terms is color coded from low (light yellow) to high (red).

Supplementary Figure 2 Directed acyclic graph (DAG) display of GO highly enriched cellular component results with candidate targeted genes. The enrichment of GO terms is color coded from low (light yellow) to high (red).

Supplementary Figure 3 Directed acyclic graph (DAG) display of GO highly enriched molecular function results with candidate targeted genes. The enrichment of GO terms is color coded from low (light yellow) to high (red).

## References

[B1] Aljakna A. Choi S. Savage H. Blair R. H. Gu T. Svenson K. L. (2012). Pla2g12b and Hpn are genes identified by mouse ENU mutagenesis that affect HDL cholesterol. PLoS One 7, e43139. 10.1371/journal.pone.0043139 22912808 PMC3422231

[B2] Alwell J. S. Abdur-Rahman A. Chukwujindu N. S. (2018). Heritability estimates of egg weight and egg shell weight in Ikenne, Nigeria. Intl. J. Sci. World 6, 38–42. 10.14419/ijsw.v6i1.8677

[B3] Andersen C. J. Blesso C. N. Lee J. Barona J. Shah D. Thomas M. J. (2013). Egg consumption modulates HDL lipid composition and increases the cholesterol-accepting capacity of serum in metabokic syndrome. Lipids 48, 557–567. 10.1007/s11745-013-3780-8 23494579 PMC3869568

[B4] Baumgartner J. Koncekova Z. Benkova J. Peskovicova D. Simenovova J. Csuka J. (2008). Changes in egg quality traits associated with long-term selection for lower yolk cholesterol content in Japanese quail. Czech J. Anim. Sci. 53, 119–127. 10.17221/2715-CJAS

[B5] Benson J. D. Bensadoun A. Cohen D. (1975). Lipoprotein lipase of ovarian follicles in the domestic chicken (*Gallus* domesticus). Proc. Soc. Exp. Biol. Med. 148, 347–350. 10.3181/00379727-148-38537 235763

[B6] Bonnet A. Bevilacqua C. Benne F. Bodin L. Cotinot C. Liaubet L. (2011). Transcriptome profiling of sheep granulose cells and oocytes during early follicular development obtained by Laser Capture Microdissection. BMC Genomics 12, 417. 10.1186/1471-2164-12-417 21851638 PMC3166951

[B7] Bulut Z. Kurar E. Ozsensoy Y. Nizamlioglu M. Garip M. Yilmaz A. (2013). Determination of chromosomal regions affecting body weight and egg production in Denizli × White Leghorn F2 populations. Eurasian J. Vet. Sci. 29, 30–38.

[B8] Damsteegt E. L. Falahatimarvast A. Mccormick S. P. Lokman P. M. (2015). Triacylglyceride physiology in the short-finned eel, Angulla australis—changes throughout early oogenesis. Am. J. Physiol. Regul. Integr. Comp. Physiol. 308, R935–R944. 10.1152/ajpregu.00436.2014 25810387

[B9] Diaz F. J. (2011). Early avian follicular development is characterized by changes in transcripts involved in steroidogenesis, paracrine signaling and transcription. Mol. Reprod. Dev. 78, 212–223. 10.1002/mrd.21288 21308853

[B10] Dikmen B. Y. Sahan U. (2007). Correlations between breeder age, cholesterol content, blood cholesterol level and hatchability of broiler breeders. Br. Poult. Sci. 48, 98–103. 10.1080/00071660601161412 17364547

[B11] Ding X. Yu Y. Su Z. Zhang K. (2017). Effects of essential oils on performance, egg quality, nutrient digestibility and yolk fatty acid profile in laying hens. Anim. Nutr. 3, 127–131. 10.1016/j.aninu.2017.03.005 29767138 PMC5941116

[B12] Diouf M. N. Sayasith K. Lefebvre R. Silversides D. W. Sirois J. Lussier J. G. (2006). Expression of phospholipase A2 group IVA (PLA2G4A) is upregulated by human chorionic gonadotropin in bovine granulose cells of ovulatory follicles. Biol. Reprod. 74, 1096–1103. 10.1095/biolreprod.105.048579 16510840

[B13] Duncan R. E. Sarkadi-Nagy E. Jaworski K. Ahmadian M. Sul H. S. (2008). Identification and functional characterization of adipose-specific phospholipase A2 (AdPLA). J. Biol. Chem. 283, 25428–25436. 10.1074/jbc.M804146200 18614531 PMC2533091

[B14] Glorian M. Duplus E. Beale E. G. Scott D. K. Granner D. K. Forest C. (2001). A single element in the phosphoenolpyruvate carboxykinase gene mediates thiazolidinedione action specifically in adipocytes. Biochimie 83, 933–943. 10.1016/S0300-9084(01)01343-8 11728630

[B15] Gohin M. Bobe J. Chesnel F. (2010). Comparative transcriptomic analysis of follicle-enclosed oocyte maturational and developmental competence acquisition in two non-mammalian vertebrates. BMC Genomics 11, 18. 10.1186/1471-2164-11-18 20059772 PMC2821372

[B16] Goraga Z. S. Nassar M. K. Brockmann G. A. (2013). Quantitative trait loci segregating in crosses between New Hampshire and White Leghorn chicken lines: I. Egg production traits. Anim. Genet. 44, 62–68. 10.1111/j.1365-2052.2012.02365.x 22404354

[B17] Griffin H. D. (1992). Manipulation of egg yolk cholesterol: a physiologist’s view. Worlds Poult. Sci. J. 48, 101–112. 10.1079/WPS19920010

[B18] Gupta A. Tiwari M. Prasad S. Chaube S. K. (2017). Role of cyclic nucleotide phosphodiesterases during meiotic resumption from diplotene arrest in mammalian oocytes. J. Cell. Biochem. 118, 446–452. 10.1002/jcb.25748 27662514

[B19] Han D. (2005). Molecular approaches to understanding variation in reproductive phenotype of female zebra finches (Taeniopygia guttata). Beijing (BJ): Peking University.

[B20] Janjira S. (2017). Preliminary study: egg production performance, egg quality and blood plasma cholesterol concentration in laying hens fed dietary dried fermented ginger and/or fermented corncob powder. Food Sci. Nutr. 3, 1–5. 10.24966/FSN-1076/100016

[B21] Klkin R. G. Yan Z. Buhman K. K. Story J. A. Turek J. J. Anderson M. (1997). Reduction of egg yolk cholesterol content through inhibition of hepatic cholesterol biosynthesis and alteration of plasma VLDL composition in laying hens: comparative effects of atorvastatin, lovastatin, and simvastatin. Atherosclerosis 134, 123. 10.1016/S0021-9150(97)88669-8

[B22] Kraus T. A. Engel S. M. Sperling R. S. Kellerman L. Lo Y. Wallenstein S. (2012). Characterizing the pregnancy immune phenotype: results of the viral immunity and pregnancy (VIP) study. J. Clin. Immunol. 32, 300–311. 10.1007/s10875-011-9627-2 22198680 PMC7086597

[B23] Kurusu S. Jinno M. Ehara H. Yonezawa T. Kawaminami M. (2009). Inhibition of ovulation by a lipoxygenase inhibitor involves reduced cyclooxygenase-2 expression and prostaglandin E2 production in gonadotropin-primed immature rats. Reproduction 137, 59–66. 10.1530/REP-08-0257 19117969

[B24] Ledur M. C. Fairfull R. W. Mcmillan I. Asseltine L. (2000). Genetic effects of aging on egg production traits in the first laying cycle of white Leghorn strains and strain crosses. Poultry Sci. 79, 296–304. 10.1093/ps/79.3.296 10735193

[B25] Lee Y. Kim H. Kim M. Chun S. (2005). Control mechanisms of ovulation by pituitary adenylate cyclase-activating polypeptide. Korean J. Fertil. Steril. 32, 101–112.

[B26] Lubzens E. Bobe J. Young G. Sullivan C. V. (2017). Maternal investment in fish oocytes and eggs: The molecular cargo and its contributions to fertility and early development. Aquaculture 472, 107–143. 10.1016/j.aquaculture.2016.10.029

[B27] Newman J. W. Stok J. E. Vidal J. D. Corbin C. J. Huang Q. Hammock B. D. (2004). Cytochrome p450-dependent lipid metabolism in preovulatory follicles. Endocrinology 145, 5097–5105. 10.1210/en.2004-0710 15308618

[B28] Omole J. O. Ighodaro O. M. (2013). Comparative studies of the effects of egg yolk, oats, apple, and wheat bran on serum lipid profile of Wistar rats. ISRN Nutr. 2013, 730479. 10.5402/2013/730479 24967250 PMC4045284

[B29] Panda A. K. Reddy M. R. Rama Rao S. V. Praharaj N. K. (2003). Production performance, serum/yolk cholesterol and immune competence of White Leghorn layers as influenced by dietary supplementation with probiotic. Trop. Anim. Health Prod. 35, 85–94. 10.1023/A:1022036023325 12636363

[B30] Rath K. P. Prasanna K. M. Bandi K. M. Nrusingha C. B. (2015). Evaluation of different egg quality traits and interpretation of their mode of inheritance in white leghorn. Vet. World 8, 449–452. 10.14202/vetworld.2015.449-452 27047113 PMC4774790

[B31] Richards J. S. Liu Z. Shimada M. (2008). Immune-like mechanisms in ovulation. Trends Endocrinol. Metabol. 19, 191–196. 10.1016/j.tem.2008.03.001 18407514

[B32] Seol H. S. Sato K. Murakami H. Toyomizu M. Akiba Y. (2006). Changes in gene expression involved in energy utilization during chicken follicle development. Anim. Reprod. Sci. 95, 283–294. 10.1016/j.anireprosci.2005.09.016 16253445

[B33] Sirois J. (1994). Induction of prostaglandin endoperoxide synthase-2 by human chorionic gonadotropin in bovine preovulatory follicles *in vivo.* Endocrinology 135, 841–848. 10.1210/endo.135.3.8070377 8070377

[B34] Sreenivas D. Prakash G. M. Mahender M. Chatterjee R. N. (2013). Genetic analysis of egg quality traits in White Leghorn chicken. Vet. World 6, 263–266. 10.5455/vetworld.2013.263-266

[B35] Su Y. Sugiura K. Wigglesworth K. O’Brien M. J. Affourtit J. P. Pangas S. A. (2008). Oocyte regulation of metabolic cooperativity between mouse cumulus cells and oocytes: BMP15 and GDF9 control cholesterol biosynthesis in cumulus cells. Development 135, 111–121. 10.1242/dev.009068 18045843

[B36] Tang H. Liu Y. Li J. Li G. Chen Y. Yin Y. (2017). LH signaling induced ptgs2a expression is required for ovulation in zebrafish. Mol. Cell. Endocrinol. 447, 125–133. 10.1016/j.mce.2017.02.042 28254490

[B37] Toups M. A. Pease J. B. Hahn M. W. (2011). No excess gene movement is detected off the avian or lepidopteran Z chromosome. Genome Biol. Evol. 3, 1381–1390. 10.1093/gbe/evr109 PMC3242482 22024813

[B38] Wang Z. Meng G. Bai Y. Liu R. Du Y. Su L. (2017). Comparative transcriptome analysis provides clues to molecular mechanisms underlying blue-green eggshell color in the Jinding duck (*Anas platyrhynchos*). BMC Genomics 18, 725. 10.1186/s12864-017-4135-2 28899357 PMC5596863

[B39] Wang Z. Shang P. Li Q. Wang L. Chamba Y. Zhang B. (2017b). iTRAQ-based proteomic analysis reveals key proteins affecting muscle growth and lipid deposition in pigs. Sci. Rep. 7, 46717. 10.1038/srep46717 28436483 PMC5402282

[B40] Yadgary L. Cahaner A. Kedar O. Uni Z. (2010). Yolk sac nutrient composition and fat uptake in late-term embryos in eggs from young and old broiler breeder hens. Poultry Sci. 89, 2441–2452. 10.3382/ps.2010-00681 20952708

[B41] Yair R. Uni Z. (2011). Content and uptake of minerals in the yolk of broiler embryos during incubation and effect of nutrient enrichment. Poultry Sci. 90, 1523–1531. 10.3382/ps.2010-01283 21673168

[B42] Yang P. Tian Y. Sun G. Jiang R. Han R. Kang X. (2013). Deposition rule of yolk cholesterol in two different breeds of laying hens. Genet. Mol. Res. 12, 5786–5792. 10.4238/2013.November.22.5 24301947

[B43] Yerushalmi G. M. Salmondivon M. Yung Y. Maman E. Kedem A. Ophir L. (2014). Characterization of the human cumulus cell transcriptome during final follicular maturation and ovulation. Mol. Hum. Reprod. 20, 719–735. 10.1093/molehr/gau031 24770949

[B44] Zhang Y. (2016). Studies on chicken hatchability and its relation with egg yolk metabolites. Niedersachsen (NI): Georg-August-University Göttingen.

[B45] Zhou L. Shi Y. Guo R. Liang M. Zhu X. Wang C. (2014). Digital gene expression profiling analysis of the cholesterol-lowering effects of alfalfa saponin extract on laying hens. PLoS One 9, e98578. 10.1371/journal.pone.0098578 24886784 PMC4041749

