# Supplementary figures and images for: Genetic Parameters for Yolk Cholesterol and Transcriptional Evidence Indicate a Role of Lipoprotein Lipase in the Cholesterol Metabolism of the Chinese Wenchang Chicken
Source: Front Genet. 2019 Oct 3;10:902. doi: 10.3389/fgene.2019.00902 (PMC6786094; doi:10.3389/fgene.2019.00902)

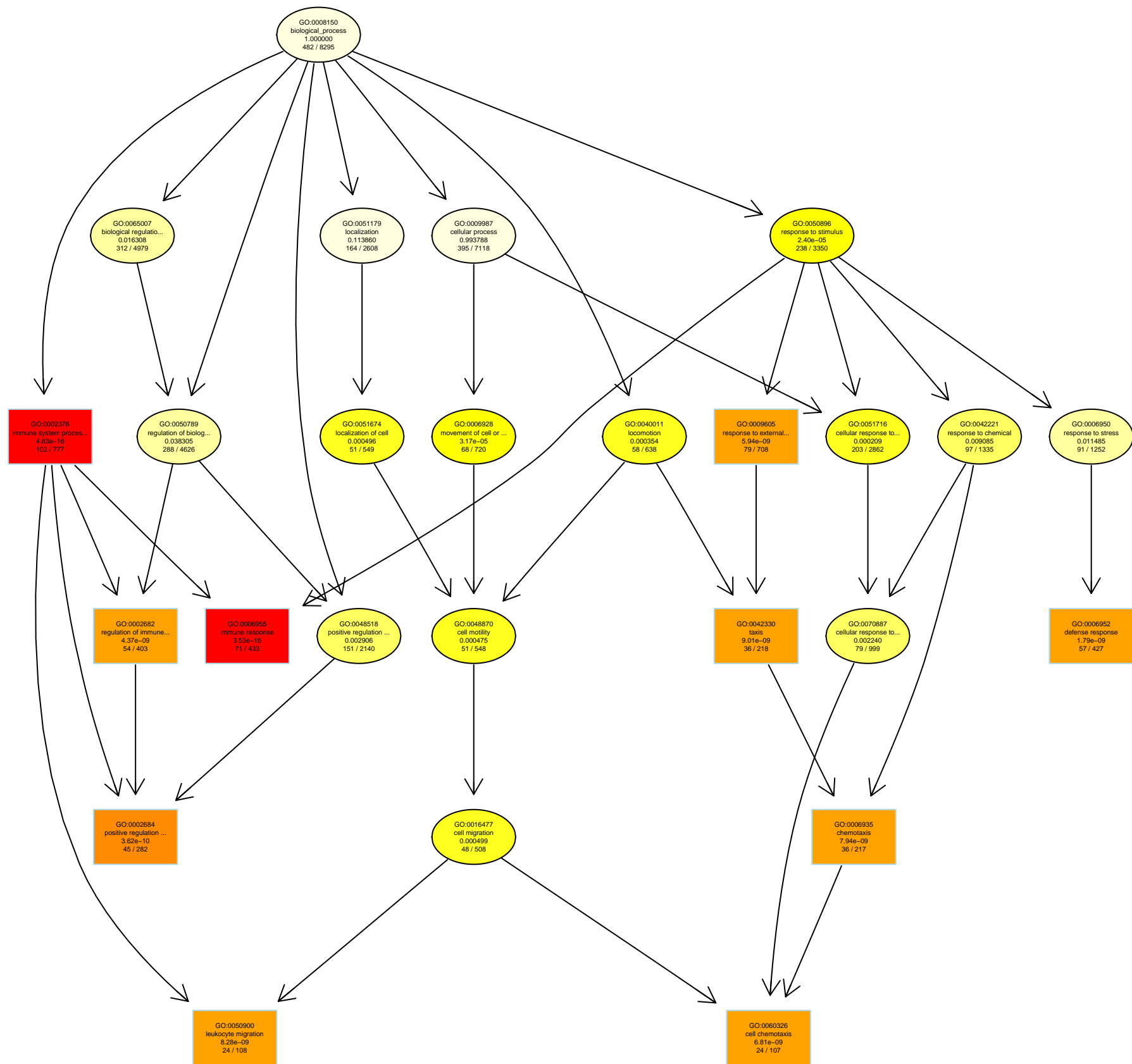

Supplement: Supplementary Figure 1 — Directed acyclic graph (DAG)display of GO highly enriched biological process results with candidate targeted genes. The enrichment of GO terms is color coded from low (light yellow) to high (red). [file Image_1.pdf]

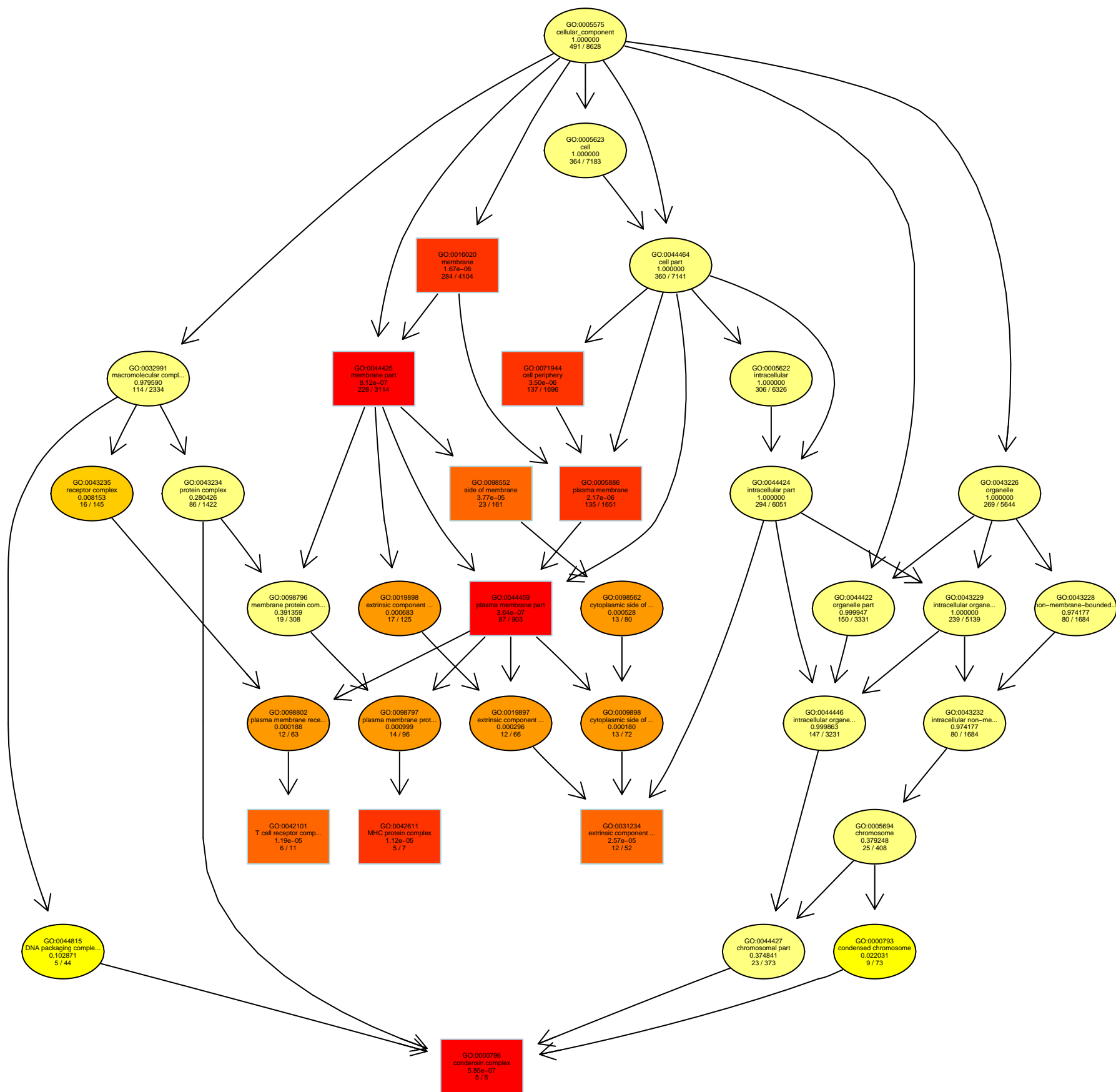

Supplement: Supplementary Figure 2 — Directed acyclic graph (DAG) display of GO highly enriched cellular component results with candidate targeted genes. The enrichment of GO terms is color coded from low (light yellow) to high (red). [file Image_2.pdf]

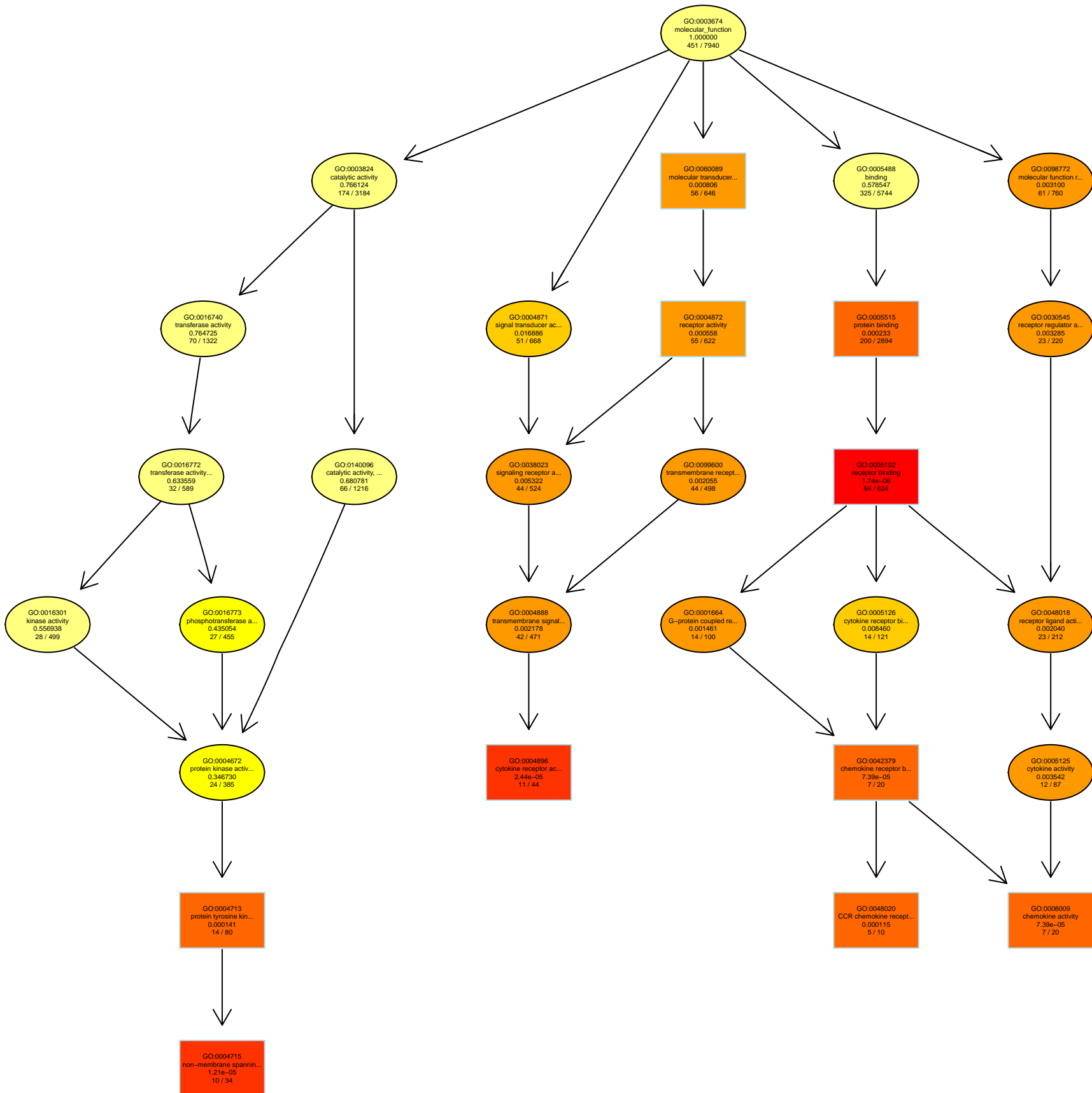

Supplement: Supplementary Figure 3 — Directed acyclic graph (DAG) display of GO highly enriched molecular function results with candidate targeted genes. The enrichment of GO terms is color coded from low (light yellow) to high (red). [file Image_3.pdf]
